# Molecular docking and pharmacokinetic studies of phytocompounds from Nigerian Medicinal Plants as promising inhibitory agents against SARS-CoV-2 methyltransferase (nsp16)

**DOI:** 10.1186/s43141-021-00273-5

**Published:** 2021-11-09

**Authors:** Tolulope Peter Saliu, Haruna I. Umar, Olawale Johnson Ogunsile, Micheal O. Okpara, Noriyuki Yanaka, Olusola Olalekan Elekofehinti

**Affiliations:** 1 Computational and Molecular Biology Unit, Department of Biochemistry, Federal University of Technology, P.M.B 704, Akure, Ondo State Nigeria; 2 Graduate School of Integrated Sciences for Life, Hiroshima University, 4-4 Kagamiyama 1-chome, Higashi-Hiroshima, 739-8528 Japan

**Keywords:** COVID-19, SARS-CoV-2, Nsp16, Phytocompounds, Pharmacokinetics

## Abstract

**Background:**

Since the index case was reported in China, COVID-19 has led to the death of at least 4 million people globally. Although there are some vaccine cocktails in circulation, the emergence of more virulent variants of SARS-CoV-2 may make the eradication of COVID-19 more difficult. Nsp16 is an S-adenosyl-L-Methionine-dependent methyltransferase that plays an important role in SARS-CoV-2 viral RNA cap formation—a crucial process that confers viral stability and prevents virus detection by cell innate immunity mechanisms. This unique property makes nsp16 a promising molecular target for COVID-19 drug design. Thus, this study aimed to identify potent phytocompounds that can effectively inhibit SARS-CoV-2 nsp16. We performed in silico pharmacokinetic screening and molecular docking studies using 100 phytocompounds—isolated from fourteen Nigerian plants—as ligands and nsp16 (PDB: 6YZ1) as the target.

**Results:**

We found that only 59 phytocompounds passed the drug-likeness analysis test. However, after the docking analysis, only six phytocompounds (oxopowelline, andrographolide, deacetylbowdensine, 11, 12-dimethyl sageone, sageone, and quercetin) isolated from four Nigerian plants (*Crinum jagus*, *Andrographis paniculata*, *Sage* plants (*Salvia officinalis L.*), and *Anacardium occidentale*) showed good binding affinity with nsp16 at its active site with docking score ranging from − 7.9 to − 8.4 kcal/mol.

**Conclusions:**

Our findings suggest that the six phytocompounds could serve as therapeutic agents to prevent viral survival and replication in cells. However, further studies on the in vitro and in vivo inhibitory activities of these 6 hit phytocompounds against SARS-CoV-2 nsp16 are needed to confirm their efficacy and dose.

**Supplementary Information:**

The online version contains supplementary material available at 10.1186/s43141-021-00273-5.

## Background

Severe acute respiratory syndrome coronavirus type 2 (SARS-CoV-2) is the most virulent human coronavirus (HCoV) possessing the ability to affect the respiratory organ and cause multi-organ failures and other related infections [1]. SARS-CoV-2 is the etiological agent for coronavirus disease 2019 (COVID-19). Since the index case of COVID-19 was reported in China, at least 187 million confirmed COVID-19 cases and over 4 million deaths have been recorded globally [2]. Lately, SAR-CoV-2 has undergone multiple mutations leading to the emergence of different variants which are more transmissible and virulent [3]. The deadlier variants of SARS-CoV-2 including the UK variant SARS-CoV-2 20I/501Y.V1, VOC 202012/01, or B.1.1.7; the South African strain SARS-CoV-2 20H/501Y.V2 or B.1.351; the Brazilian/Japanese variant SARS-CoV-2 P.1; and the other emerging variants are complicating the global burden of COVID-19 [4, 5].

Generally, coronaviruses have the largest genomes of all RNA viruses with approximately 29,800 bases that encode 4 structural proteins, 9 accessory proteins, and 16 non-structural proteins (nsp) numbered from nsp1-16 which are essential for the viral life cycle [6–8]. Although these proteins have been explored as therapeutic targets for COVID-19 drugs, most of the drugs are not without some side effects. Hence, there is an urgent need to take a parallel and multidirectional approach to counter the spread of SARS-CoV-2. Consequently, an extensive exploration of natural sources for therapeutic compounds which target SARS-CoV-2 protein(s) and have minimal or no side effects on humans become essential.

The distinguishing feature between eukaryotic and viral mRNAs is the presence of a 5′ cap in the former which confers stability on the eukaryotic mRNA. Thus, for SARS-CoV-2 to survive inside their host, they must develop a modification system to cap their RNAs at the 5′ end. The enzyme that mediates this capping in a methylation reaction is an S-adenosyl-L-Methionine-dependent methyltransferase which in the case of coronavirus is nsp16. Methylation enables the virus to mimic the host’s mRNA structure thereby protecting the viral mRNA from degradation by the host’s 5′–3′ exoribonucleases [9]. As a result, the viral mRNA can escape recognition and targeting by the immune response thereby allowing efficient translation of the mRNA and subsequent production of virion particles. Notably, nsp16 is only active in the presence of its binding partner, nsp10, which is involved in the N-7 methylation of GTP nucleobase. These two proteins form an nsp16:nsp10 complex that is very crucial for the replication process of SARS-CoV-2 [10]. However, nsp16 has been recognized as a more promising and indispensable molecular target for therapeutic agents against COVID-19 [11]. More so, it has been shown that the substitution of a conserved region KDKE of nsp16 is sufficient to attenuate viral infection in vitro and in vivo [11, 12].

In this study, in silico virtual screening for potential drug candidates against coronavirus nsp16 was conducted with 100 compounds isolated from some Nigerian medicinal plants reported to possess antiviral properties.

## Methods

### Protein target selection and preparation

**Fig. 1:**
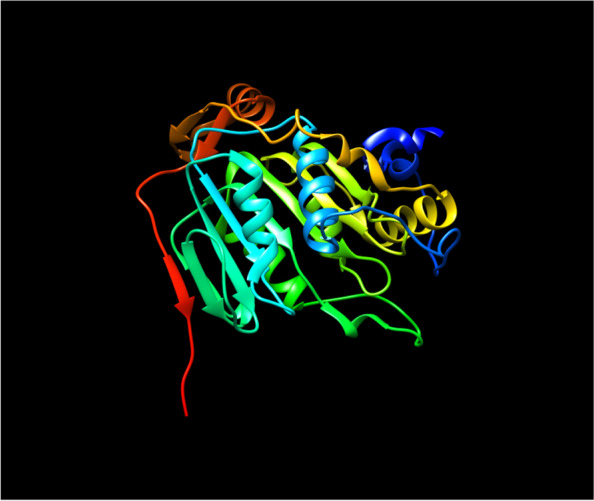
3D structure of nsp16 free from nsp10 and Sinefungin

The 3-dimensional (3D) X-ray crystallographic structure of SARS-CoV-2 nsp10-nsp16 methyltransferase complex with Sinefungin (SFG) (PDB ID: 6YZ1) solved at 2.4 Å resolution was retrieved from RCSB protein data bank (PDB) (https://www.rcsb.org/structure/6YZ1) (Fig. 1). The protein was prepared for docking by the following steps: (a) nsp10 protein was removed, (b) metal ions, water, and cofactors were removed, (c) bounded ligand SFG was removed, (d) polar hydrogen bond was added, and (e) finally, the nsp-16 protein was minimized using the relevant tools in Cresset Flare© software, version 4.0 (https://www.cresset-group.com/flare/). The protein minimization was based on the General Amber Force Field (GAFF), with a gradient cutoff of 0.200 kcal/mol/A, and iterations were set to 2000 iterations [13].

#### Ligand selection and preparations

 A total of 100 phytocompounds isolated from fourteen Nigerian-based plants that have been previously reported to have antiviral activities were investigated. The plants—including Sage plants (*Salvia officinalis L.*), *Borreria verticillate*, *Sida cordifolia*, *Licorice* (*Glycyrrhiza glabra*), *Crinum jagus*, *Andrographis paniculate*, *Phyllanthus amarus*, *Echinacea Purpurea*, *Anacardium occidentale*, *Khaya grandifoliola*, *Detarium microcarpum, Sterculia setigera,* and *Piliostigma thonningii*—were selected for virtual screening and molecular docking study against SARS-CoV-2 nsp16 [14–20]. The 3D structures of most of the phytocompounds were obtained from the PubChem database (https://pubchem.ncbi.nlm.nih.gov/) in simple document format (SDF) while the structures of others were drawn using MarvinSketch© (ver. 15.11.30). All the phytocompounds were optimized using Open Babel in Python Prescription (version 0.8) which converted the ligands to the most stable structures energetically using Merck molecular force field (MMFF94). The names of all the phytocompounds selected and their source plants are given in Table 1.

**Table 1:** List of phytocompounds (numbered 1–100) obtained from 14 Nigerian medicinal plants having antiviral properties

S/N	Name of medicinal plant with antiviral properties	Major phytocompounds selected
1	Sage plants (*Salvia officinalis L*.)	(1) Safficinollide, (2) **Sageone**, (3) **11,12-dimethyl sageone**
2	*Borreria verticillate*	(4) Verticillatine A, (5) Verticillatine B, (6) Scandoside methyl ester
3	*Sida cordifolia*	(7) B-phenethylamine, (8) Hypaphorine, (9)Vasicine, (10) Vasicinone, (11) Vasicinol, (12) Cryptolepine, (13) Malvalic acid, (14) Sterculic Acid, (15) 5,7-dihydroxy-3-isoprenyl flavones, (16) 5-dihydroxy-3-isoprenyl flavones, (17) 20-hydroxyecdysone, (18) 20-hydroxy-(25-acetyl)-ecdysone-3-O-β-D-glucopyranoside, (19) Sidasterone A,(20) Sidasterone B,(21) S-(+)-N b-methyltryptophan methyl ester, (22) 5`-hydroxymethyl-1`-(1,2,3,9-tetrahydro-pyrrolo [2, 1-b] quinazoline-1-yl)-hepta-1-one)
4	Licorice (*Glycyrrhiza glabra*)	(23) Licochalcone A, (24) Licochalcone E, (25) Glabridin, (26) Glycyrrhetinic acid, (27) Liquiritigerin
5	*Crinum jagus*	(28) Androlycorine, (29) Dihydrolycorine, (30) Vittatine (31) 8-O-demethylmaritidine, (32) Powelline, (33) **Oxopowelline,** (34) Buphanidrine, (35) Galanthamine, (36) Sanguinine, (37) Narwedine, (38) **Deacetylbowdensine**, (39) Undulatine, (40) Galanthamine-N-Oxide, (41) Lycorine
6	*Andrographis paniculata*	(42) **Andrographolide**, (43) Andrograpanin
7	*Phyllanthus amarus*	(44) Gallocatechin, (45) 4-O-Galloylquinic acid, (46) Corilagin, (47) Isocorilagin, (48) Phyllanthine, (49) Securinine, (50) Isobubbialine, (51) Epibubbialine, ( 52)Nor securinine, (53) Oleanolic acid (54) Ursolic acid, (55) Linalool, (56) Amarosterol A, (57) Amarosterol B, (58) Phyllanthenol, (59) Phyllantheol, (60) Lupeol, (61) Ellagic Acid, (62) Gallic acid, (63) Phytol
8	*Echinacea purpurea*	(64) Cichoric Acid, (65) Chlorogenic Acid, (66) Caffeic Acid, (67) Nitidarin diisovalerianate
9	*Anacardium occidentale*	(68) Stigmasterol, (69) (E)-caryophyllene, (70) Sitosterol 3-O-β-galactopyranoside, (71) Sitosterol, (72) Germacrene D, (73) Quercetin 3-O-rhamnoside, (74) **Quercetin**, (75) Isoquercetrin, (76) Rutin
10	*Khaya grandifoliola*	(77) Deacetylkhayanolide E, (78) Khayanolide A, (79) 6-Phenyl,4-(1’oxyethylphenyl) hexane, (80) Benzene 1,1’-(oxydiethylidene) bis, (81) Carbamic acid, 4-methyl-1-phenyl)-1-phenyl
11	*Detarium microcarpum*	(82) 3,4-Epoxyclerodan-13E-en-15-oic acid, (83) 5α,8α-(2-oxokolavenic acid), (84) Copalic acid, (85) 3,4-dihydroclerodan-13z-en-15-oic acid, (86) 3,4-dihydroxyclerodan-13E-en-15-oic acid, (87) Oxokolavemic acid
12	*Sterculia setigera*	(88) 3,4-Dimethoxyphenol β-D-apiofuranosyl(1′->6′)-β-D-glucopyranoside, (89) Procyanidin B2
13	*Piliostigma thonningii*	(90) 2β-methoxyclovan-9α-ol, (91) Methyl-ent-3β-hydroxylabd-8(17)-en-15-oate, (92) Clovane-2β,9α-diol, (93) Alepterolic acid, (94) Anticopalic acid, (95) (3S,5R,6S)-trihydroxy-7E-megastigmen-9-one, (96) β-amyrin, (97) Piliostigmin, (98) Vitamin E, (99) 3-hexenyl-1-O β-D-glucopyranoside
14	*Detarium senegalense*	(100) Anthocyanidin alkaloid

### Screening of compounds for drug-likeness

The selected phytocompounds were screened for drug-likeness and medicinal properties were predicted with the aid of SwissADME web tool (www.swissadme.ch/index.php) [21]. The canonical SMILES of these compounds were uploaded on the server and run to predict their drug-likeness using several scoring schemes which included Lipinski’s rule of five, Ghose’s filter, Veber’s rule, Egan’s rule, and Muegge’s rules [22–26].

### Molecular docking validation

To substantiate the accuracy and reliability of the docking results, the docking protocol was validated according to our previous works [27, 28] which was based on the method by Warren et al. [29]. The purpose was to regenerate the binding pose and the molecular interaction of the co-crystalized ligand of the experimentally crystalized protein structure accurately. Thereupon, the native ligand Sinefungin (SFG) of the X-ray protein was detached from the protein and then prepared for docking in Cresset Flare© software, version 4.0 (https://www.cresset-group.com/flare/). The ligand was then re-docked back into the active site of nsp16 using AutoDock Vina in PyRx [30]. The docked complex was superimposed onto the X-ray resolved crystal of nsp16 bearing the co-crystalized ligand to compute the root mean square deviation (RMSD) value in PyMOL. The interaction analysis of both complexes was then evaluated using LigPlot^+^ software [31].

### Molecular docking

Molecular docking was accomplished via a flexible docking protocol [30]. Briefly, Python Prescription 0.8, a suite housing the AutoDock Vina module, was employed for the molecular docking study of 59 phytocompounds with SARS-CoV-2 nsp16. The specific target site for the receptor was set using the grid box with dimensions (21.6286 × 26.8772 × 20.7369) Å, and the center was adjusted based on the active site of the enzyme which consists of the following amino acids: Tyr47, Asn43, His69, Asp99, Asn101, Asp114, Asp130, and Lys170. At the end of the docking experiment, phytocompounds with docking scores above the control Sinefungin (SFG) were subjected to molecular interaction analysis with the aid of PyMOL© Molecular Graphics (version 2.4, 2016, Shrodinger LLC) and Discovery Studio 2016©.

### ADMET property prediction of active phytocompounds

The ADMET (absorption, distribution, metabolism, excretion, and toxicity) properties of the hit phytocompounds obtained from our virtual screening were analyzed using admetSAR web server (http://lmmd.ecust.edu.cn/) [32]. The 3D structures of the top 6 phytocompounds were saved in canonical SMILES format and were uploaded on admetSAR web server. The predicted pharmacokinetic parameters which influence drug absorption include human intestinal absorption (HIA), blood-brain barrier permeation, and the likeliness of being P-glycoprotein substrate [33, 34]. The ability to inhibit different CYP450 enzymes or the likeliness of being a substrate to CYP450 enzymes [35, 36] are the significant properties predicted to influence phytochemical metabolism. Finally, toxicity predictions were performed based on several computational models which included the Ames test for mutagenicity and carcinogenicity [32, 37].

## Results

### Drug-likeness analysis of selected phytocompounds

A drug-likeness analysis is an important segment of drug development that is used to identify the biological properties of drug candidates. We used SwissADME web tool to evaluate the drug-likeness properties of 100 phytocompounds isolated from fourteen Nigerian-based plants (Table 1). We found that 59 out of 100 phytocompounds (Supplimentary Table 1) tested satisfied all evaluated drug-likeness scoring schemes namely: Lipinski’s rule of five, Ghose’s filter, Veber’s rule, Egan’s rule, and Muegge’s rules [22–26]. Thus, this filtered list of 59 phytocompounds was designated as druggable and was subsequently used for further analysis.

### Molecular docking validation

**Fig. 2:**
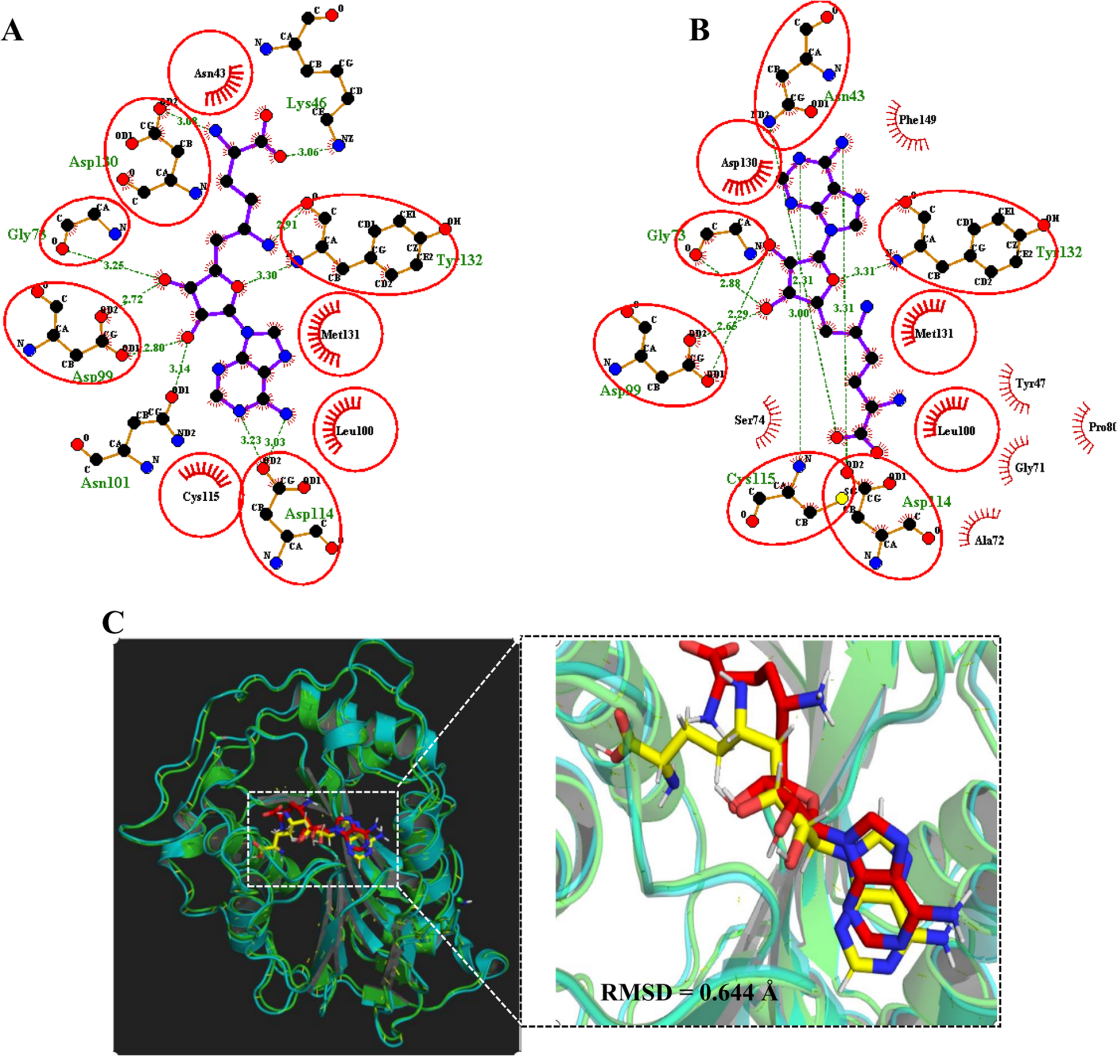
2D molecular interaction analysis and superimposition of re-docked nsp16. **A** 2D molecular interaction of re-docked SGF with nsp16 active site. **B** The binding sites of SGF with nsp16 active site in co-crystalize complex. **C** Superimposition of the co-crystallized ligand (yellow) and the re-docked ligand (Red) (RMSD = 0.644 Å)

To validate the docking procedure and to eliminate false-positive results, two different methodologies were used namely re-docking and superimposition. After SFG (an inhibitor of nsp16) was removed and re-docked into the active site using AutoDock Vina in PyRx [30], we found that the inhibitor bound exactly to the active site with a binding energy of − 7.9 kcal/mol. The re-docked complex was then found to interact with the same amino acid residues (Asn43, Gly73, Leu100, Asn101, Asp114, Cys115, Tyr132, Asp130, Met131, and Asp99) compared to the native co-crystallized complex (Fig. 2A, B) [11]. Subsequently, using PyMOL, the re-docked nsp16:SFG complex was superimposed onto the native co-crystallized nsp16:SFG from PDB and the RMSD was calculated. Our result showed a low RMSD of 0.644 Å (Fig. 2C). This partially suggests that the docking protocol was efficient and valid [38, 39].

#### Molecular Docking

**Fig. 3:**
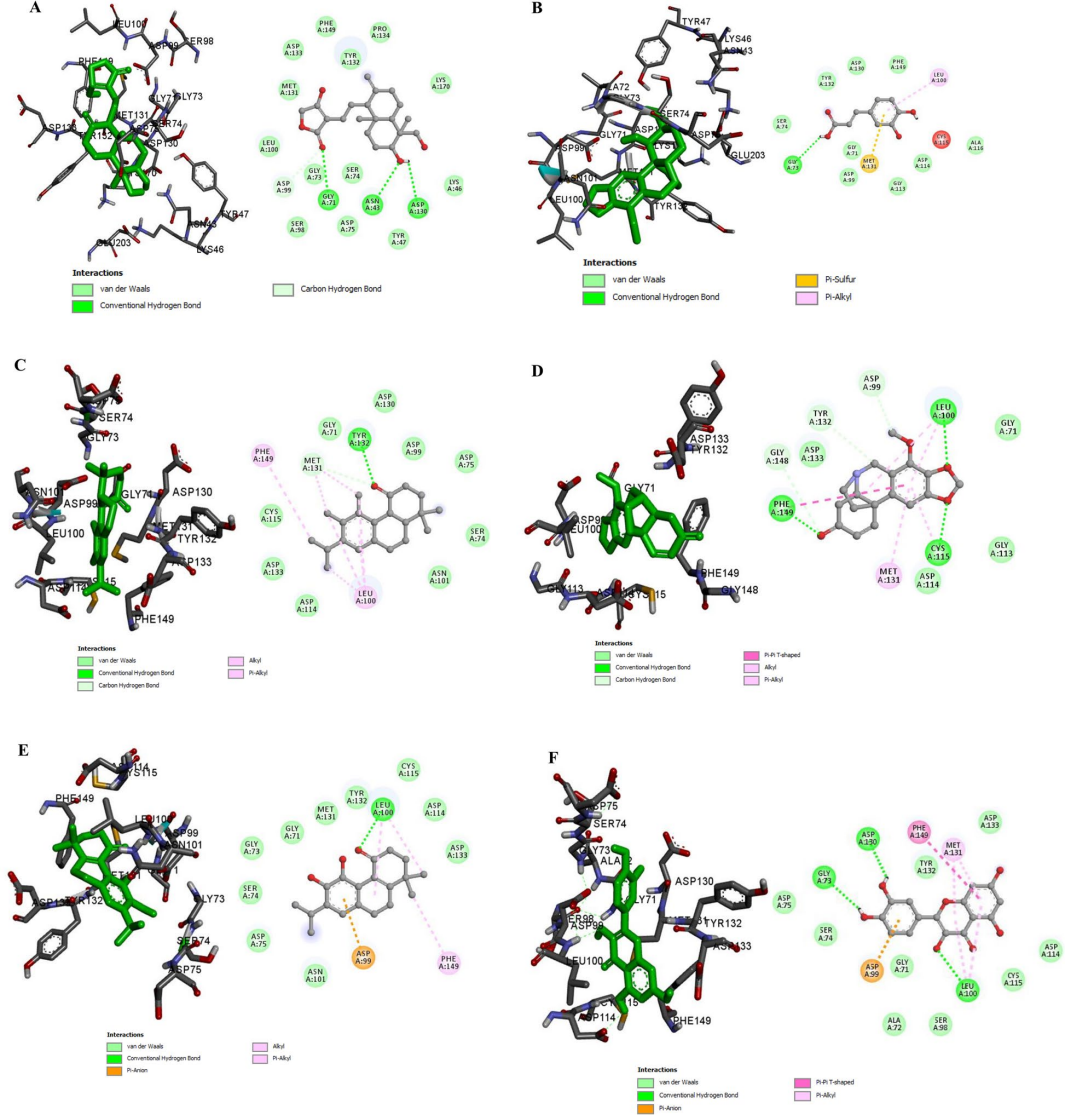
2D and 3D binding mode of best 6 phytocompounds with the active site of nsp16. **A** Andrographolide. **B** Deacetylbowdensine. **C** 11, 12-dimethylsageone. **D** Oxopowelline. **E** Sageone. **F** Quercetin. From the 3D presentation, ligands are colored in green sticks while amino acid residues are in black sticks. Gray sticks and spheres represent the ligands and amino acid residues respectively in 2D presentation

**Fig. 4:**
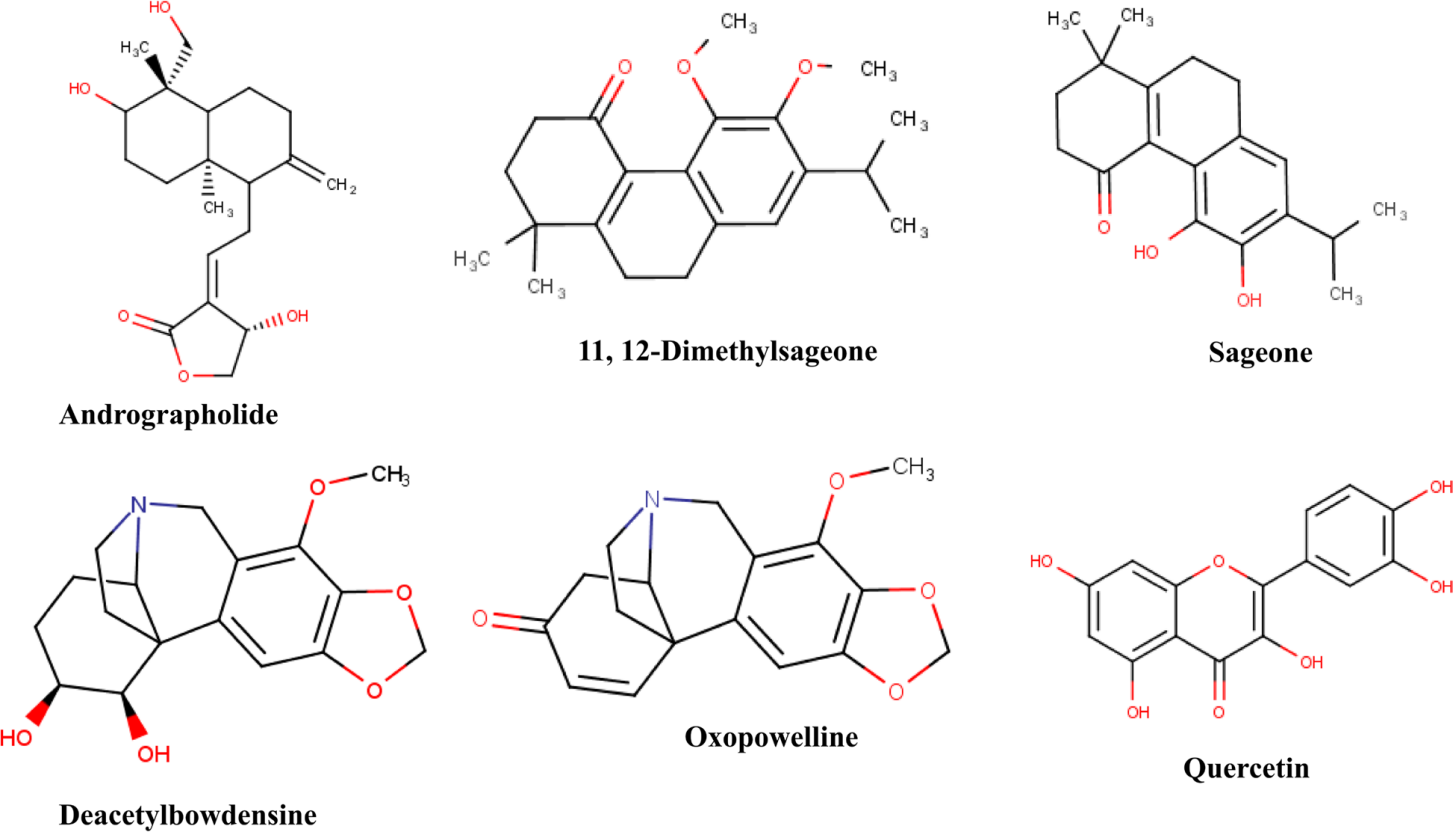
2D structure of 6 hit phytocompounds with a high binding energy

The molecular docking of all 59 phytocompounds that passed the drug-likeness test was performed on SARS-CoV-2 nsp16 (PDB ID: 6YZ1) using AutoDock Vina in PyRx [30]. We analyzed the active phytocompounds by binding free energies score and molecular interaction profile. Out of the 59 phytocompounds, only 6 (oxopowelline, andrographolide, deacetylbowdensine, 11, 12-dimethyl sageone, sageone, and quercetin) displayed the best binding affinity (ranging from − 7.9 to − 8.4 kcal/mol) and interactions (Tables 2 and 3, Fig. 3). The 2D structures of the 6 hit phytocompounds which displayed very efficient binding with nsp16 are shown in Fig. 4. The remaining 53 phytocompounds did not show efficient binding score; thus, they were not pursued further.

**Table 2:** Binding affinity of compounds from antiviral plants when docked against nsp16

S/N	Compounds	Binding affinity (kcal/mol)
1.	Compound 1	−5.9
2.	1-O-Caffeoylglycerol	−6.8
3.	1-O-pCoumaric acid	−6.1
4.	compound 3	−5.9
5.	Compound 4	−7.2
6.	5-dihydroxy-3-isopreyl flavones	−7.4
7.	5′-hydroxymethyl-1′-(1,2,3,9-tetrahydro	−6.7
8.	compound 6	−6.0
9.	Compound 9	−7.8
10.	Compound 2	−7.4
11.	Compound 14	−6.2
12.	3,4-dihydroxyclerodan-13E-en-15-0ic acid	−7.0
13.	5,7-dihydroxy-3-isoprenyl flavones	−7.7
14.	11,12-dimethyl sageone*	−**8.1**
15.	Anthocyanidin alkaloid	−6.5
16.	Benzene 1,1′-(oxydiethylidene) bis	−5.9
17.	Caffeic Acid	−6.2
18.	(4-methyl-1-phenyl)-1-phenyl Carbamic acid	−5.8
19.	B-phenethylamine	−4.6
20.	Vasicine	−6.1
21.	Vasicinol	−6.4
22.	Buphanidine	−7.3
23.	Vasicinone	−6.7
24.	Sterculic acid	−4.9
25.	Vittatine	−7.2
26.	Sanguinine	−7.0
27.	Securinine	−6.7
28.	Powelline	−7.3
29.	Asperuloside	−7.4
30.	Glabridin	−7.8
31.	Phyllantine	−6.6
32.	8-O-demethylmaritidine	−6.7
33.	Oxopowelline*	−**7.9**
34.	Deacetylbowdensine*	−**8.0**
35.	Andrlycorine	−7.2
36.	Hypaphorine	−6.3
37.	3,4-Epoxyclerodan-13E-en-15-oic acid	−6.9
38.	Andrograpanin	−7.1
39.	5alpha,8alpha-(2-oxokolavenic acid)	−6.5
40.	Epibubbialine	−6.3
41.	Copalic acid	−7.1
42.	Dihydrolycorine	−7.8
43.	Galanthamine	−6.5
44.	Narwedine	−7.1
45.	Undulatine	−7.2
46.	Sageone*	−**8.1**
47.	Scafficinolide	−6.8
48.	Licochelcone A	−7.3
49.	Licochelcone E	−7.0
50.	Andrographolide*	−**7.9**
51.	Isobubbialine	−6.0
52.	Verticillatine A	−5.9
53.	Ellagi acid	−7.8
54.	Liquiritigerin	−7.8
55.	Lycorine	−7.8
56.	Oxokolavemic acid	−7.1
57.	p-Coumaric acid	−5.3
58.	Quercetin*	−**8.4**
59.	S-(+)-N b-methyltryptophan methyl ester	−6.1
60.	SFG^#^	−**7.9**

*Phytocompounds with the best binding affinity. ^#^Reference compound

**Table 3:** Binding affinity and molecular interactions of the six hit compounds when docked against nsp16

S/N.	Compounds	Binding affinity (kcal/mol)	Hydrogen bond interactions (distance)	Hydrophobic interactions	Electrostatic interactions
1	Quercetin	−8.4	Asp130 (3.26), Gly73 (4.10) and Leu100 (5.10)	Met131, Phe149, Tyr132, Glu71, Asp75, Ser74, Ala72, Ser98, Leu100, Cys115, Asp114 and Asp133	Asp99, π-Anion
2	Sageone	−8.1	Leu100 (4.25)	Met131, Phe149, Tyr132, Gly73, Gly71, Asp75, Ser74, Ala72, Ser98, Leu100, Cys115, Asp114 and Asp133	Asp99, π-Anion
3	11, 12-Dimethylsageone	−8.1	Met131 (4.72) and Tyr132 (5.72)	Asn101, Ser74, Leu100, Asp114, Asp133, Cys115, Phe149, Met131, Gly71, Asp130, Asp99 and Asp75	–
4	Deacetylbowdensine	−8.0	Gly73 (3.70)	Ser74, Tyr132, Asp130, Phe149, Leu100, Cys115, Ala116, Asp114, Gly113, Met131, Gly71 and Asp99	–
5	Oxopowelline	−7.9	Phe149 (4.42), Leu100 (4.66), Cys115 (3.72), Gly148 (4.11), Tyr132 (6.94) and Asp99 (5.14)	Phe149, Met131, Cys115, Asp114, Gly113, Gly71 and Asp133	–
6	Andrographolide	−7.9	Gly71 (3.59), Asp99 (3.45), Asn43 (4.44) and Asp130 (4.76)	Cys46, Lys170, Tyr47, Asp75, Ser98, Leu100, Met131, Asp133, Phe149, Tyr132, Pro134 and Ser74	–

#### ADMET properties prediction of active phytocompounds

To reduce the high rate of attrition of candidates from the drug development pipeline, it is crucial to evaluate the ADMET properties of drug candidates. Therefore, the top 6 phytocompounds were subjected to ADMET properties prediction using the admetSAR web server [32]. The ADMET parameters predictions indicated that the 6 phytocompounds identified as potent nsp16 inhibitors abide by the pharmacokinetics rules and showed minimal cytotoxicity. As shown in Table 4, the prediction of HIA, the likeliness of being P-glycoprotein substrate, blood-brain barrier permeability, Ames mutagenesis, carcinogenicity, or aqueous solubility suggest that the 6 hit phytocompounds have suitable drug profile.

**Table 4:** ADMET profiling of compounds with the best hit

ADMET models	Oxopowelline	Deacetylbowdensine	Sageone	Andrographolide	11, 12-Dimethyl sageone	Quercetin	SFG
**Ames mutagenesis**	−	−	−	−	−	+	−
**Acute oral toxicity (c)**	III	III	III	III	III	II	III
**Androgen receptor binding**	−	+	+	+	+	+	−
**Aromatase binding**	−	−	+	+	−	+	−
**Avian toxicity**	−	−	−	−	−	−	
**Blood-brain barrier**	+	+	+	+	+	−	−
**BRCP inhibitor**	−	−	−	−	−	+	
**Biodegradation**	−	−	−	−	−	−	−
**BSEP inhibitor**	+	+	−	+	+	−	
**Caco-2**	+	+	+	+	+	−	−
**Carcinogenicity**	−	−	−	−	−	−	−
**CYP1A2 inhibition**	−	−	+	−	−	+	−
**CYP2C19 inhibition**	−	−	+	−	−	−	−
**CYP2C9 inhibition**	−	−	−	−	−	−	−
**CYP2C9 substrate**	−	−	−	−	−	−	−
**CYP2D6 inhibition**	+	−	−	−	−	−	−
**CYP2D6 substrate**	−	+	−	−	−	−	−
**CYP3A4 inhibition**	+	+	−	−	−	+	−
**CYP3A4 substrate**	+	+	+	+	+	+	−
**CYP inhibitory promiscuity**	−	−	−	−	−	+	−
**Eye corrosion**	−	−	−	−	−	−	−
**Eye irritation**	−	−	−	−	−	+	−
**Estrogen receptor binding**	+	+	+	+	+	+	−
**Fish aquatic toxicity**	+	−	+	+	+	+	
**Glucocorticoid receptor binding**	+	+	+	+	+	+	
**Honey bee toxicity**	+	+	+	+	+	+	
**Hepatotoxicity**	+	−	−	−	−	+	−
**Human either-a-go-go inhibition**	−	−	−	−	+	−	−
**Human intestinal absorption**	+	+	+	+	+	+	−
**Human oral bioavailability**	−	−	+	−	+	−	−
**MATE1 inhibitor**	−	−	−	−	−	+	
**Acute oral toxicity**	2.36	2.73	1.5	2.79	1.36	2.56	1.19
**OATP1B1 inhibitor**	+	+	+	+	+	+	
**OATP1B3 inhibitor**	+	+	+	+	+	+	
**OATP2B1 inhibitor**	−	−	−	−	−	+	
**OCT1 inhibitor**	+	−	+	−	+	−	
**OCT2 inhibitor**	−	−	−	−	−	−	
**P-glycoprotein inhibitor**	−	−	−	−	−	−	−
**P-glycoprotein substrate**	−	−	−	−	−	−	+
**PPAR gamma**	+	+	+	−	+	+	−
**Plasma protein binding**	0.72	0.89	1.17	0.54	1.17	1.17	7.61
**Subcellular localization**	Mitochondria	Lysosomes	Mitochondria	Mitochondria	Mitochondria	Mitochondria	Nucleus
**Tetrahymena pyriformis**	1.67	1.17	1.43	0.8	1.46	0.89	2.66
**Thyroid receptor binding**	−	+	+	+	+	+	
**UGT catalyzed**	−	+	+	+	−	+	
**Water solubility**	−2.55	−2.22	−4.84	−2.85	−4.72	−3	−0.73

## Discussion

The current treatment strategy that has been employed globally to combat COVID-19 involves the use of different vaccine cocktail which were developed from four major structural proteins (nucleocapsid protein, spike glycoprotein, membrane glycoprotein, and small envelope glycoprotein) of SARS-CoV-2. Most of these vaccines have demonstrated between 90 and 95% efficacy against SARS-CoV-2 [40, 41]. However, with the emergence of several mutated variants of SARS-CoV-2, the efficacy of the current vaccine therapy may be reduced. Hence, this necessitates an urgent search for new therapeutic strategies to treat COVID-19. The non-structural protein, nsp16, is an important 2’-O-methyltransferase enzyme that is critical for converting viral mRNA cap-0 into cap-1 structure—an essential process that prevents virus detection by the host’s cell innate immunity mechanisms [9, 39, 42, 43]. Targeted inhibition of nsp16, which plays a key role in viral RNA stability and life cycle, would be a valuable strategy for COVID-19 therapeutic intervention [11]. In the present study, we used in silico pharmacokinetic screening and molecular docking studies to discover 6 phytocompounds (oxopowelline, andrographolide, deacetylbowdensine, 11, 12-dimethyl sageone, sageone, and quercetin) isolated from four Nigerian plants (*Crinum jagus*, *Andrographis paniculata*, Sage plants (*Salvia officinalis L.*), and *Anacardium occidentale*) which showed potential to inhibit SARS-CoV-2 nsp16.

Generally, phytocompounds isolated from numerous medicinal plants have been shown to be potential drug candidates for various viral and respiratory diseases [44]. For example, emodin (a phytocompound isolated from *Rheum emodi*) has been reported to significantly inhibit the 3a ion channel of SARS-CoV and HCoV-OC43 as well as virus release from HCoV-OC4 [45]. In a different study, Ho et al. showed that emodin inhibited the direct binding of SARS-CoV S protein to ACE2 and S protein-pseudo-typed retrovirus [46]. Based on these reports, the search for phytocompounds from medicinal plant targeting key proteins/enzymes of SARS-CoV-2 could lead to the discovery of new therapeutic agents for treating COVID-19 disease. Thus, in this study, 100 phytocompounds from fourteen Nigerian medicinal plants, having antiviral properties, were subjected to drug-likeness analysis. This analysis ensured the removal of phytocompounds with poor pharmacokinetic parameters from the drug pipeline, thus saving time and cost [47]. Using SwissADME web tool, we found that 59 of the 100 phytocompounds screened are potentially druggable based on multiple scoring schemes.

Once we identified the phytocompounds that met the drug-likeness criteria, we used AutoDock Vina program in PyRx [30] to predict the possible binding affinity against SARS-CoV-2 nsp16. The docking score (− 7.9 kcal/mol) and binding pose of SFG were used as a reference [11, 48]. By applying SFG as a reference compound, the molecular docking study revealed that out of the 59 phytocompounds screened, only six (oxopowelline, andrographolide, deacetylbowdensine, 11, 12-dimethyl sageone, sageone, and quercetin) showed efficient binding affinities with the active site of SARS-CoV-2 nsp16 (Tables 3 and 4). The top six phytocompounds identified were isolated from four (*Crinum jagus*, *Andrographis paniculata*, Sage plants (*Salvia officinalis L.*), *Anacardium occidentale*) out of the fourteen screened Nigerian medicinal plants with antiviral properties [14, 17–19, 49–52]. Oxopowelline and deacetylbowdensine with respective binding energies of − 7.9 kcal/mol and − 8.0 kcal/mol were isolated from *Crinum jagus*. Andrographolide with binging energy of − 7.9 kcal/mol was isolated from *Andrographis paniculate*. 11, 12-dimethyl sageon and sageon with binding energies of − 7.9 kcal/mol and − 8.0 kcal/mol respectively were isolated from Sage plants (*Salvia officinalis L.*) while quercetin which possess the highest binding energy of − 8.4 kcal/mol was isolated from *Anacardium occidentale*. Although not so much investigation on the anti-SARS-CoV-2 activity of oxopowelline, deacetylbowdensine, 11, 12-dimethyl sageon, and sageon have been done, there are evidence that indicate that andrographolide and quercetin may be therapeutic agents against COVID-19. Following an in vitro study, Shi et al. reported that andrographolide and its fluorescent derivative (nitrobenzoxadiazole-conjugated andrographolide) inhibited the activities of both SARS-CoV and SARS-CoV-2 main protease (M^pro^). In a different study, Chen et al. showed that a quercetin derivative (quercetin-3-b-galactoside) inhibited the activity of SARS-CoV 3C-like protease (3CL^Pro^) [53, 54]. Previous study has shown that among the amino acid residues present in the active site of SARS-CoV-2 nsp16, six residues (Asp99, Asn101, Asn43, Asp130, and Lys170 and Asp114) are absolutely conserved [11]. Therefore, to prevent RNA methylation for coronaviruses, it is critical to prevent the interaction of any of these residues with the substrate (S-adenosylmethionine (SAM)). Interestingly, all the six phytocompounds can form hydrogen interaction with some of these residues.

Besides targeting SARS-CoV-2 nsp16, the 6 phytocompounds identified have been suggested as potential candidates with antibacterial, anti-inflammatory, antioxidant, antiviral activity, acetylcholinesterase (AChE), or butyrylcholinesterase (BChE) inhibitors [53–56]. Oxopowelline and deacetylbowdensine have recently been shown to be potential inhibitors of these two enzymes (BChE and AChE) [57]. Therefore, the inhibition of cholinesterase activities could be an additional strategy used by oxopowelline and deacetylbowdensine to prevent chronic cytokine storm associated with individuals with severe COVID-19. Furthermore, it is worth mentioning that the four plants containing the 6 identified phytocompounds have been reported to have other pharmacological effects that can be highly beneficial in the treatment of COVID-19 symptoms such as cough, fever, muscle pain, chest pain, abdominal pain, and diarrhea [14, 17–19, 49–52]. In line with this, *Andrographis paniculate* and *Anacardium occidentale* have been reported to be traditionally used for the treatment of diarrhea and fever. Also, an in vivo study demonstrated that *Salvia officinalis L.* has analgesic and anti-inflammatory effects in mice and rats [58] suggesting the possibility of being used to alleviate the pain associated with COVID-19. A recent study has also demonstrated that *Crinum jagus* and *Anacardium occidentale* were some of the most popular plants used against cough, asthma, and other respiratory conditions in Nigeria [59], thus indicating that these plants may be highly effective in the management of cough which is associated with COVID-19 disease.

Finally, we examined the ADMET properties of the 6 phytocompounds to validate their pharmacokinetic potential against COVID-19 since a poor ADMET profile is sufficient to prevent therapeutic agents from getting clinical approval [60]. The ADMET profile result given in Table 4 indicated that none of the 6 compounds could be carcinogenic or inhibit p-glycoprotein. The HIA of all the 6 phytocompounds were favorable; however, only quercetin could be mutagenic and cross the blood-brain barrier. Interestingly, the benefits of quercetin was reported in some clinical trials and reviewed by Okamoto [61]. Similarly, clinical trial data on the use of andrographolide as treatment for acute and chronic throat and respiratory disease showed that it has no significant adverse effect in the patients [62]. Apart from oxopowelline and quercetin, all the other 4 phytocompounds showed no hepatotoxicity. The water solubility ranged between −4 and −2, an indication that each of the 6 phytocompounds was either moderately soluble or highly soluble. Although our data indicated that quercetin could cross the blood-brain barrier, this does not nullify its potential as a drug candidate. T*he ability of quercetin to* cross the *blood*-*brain barrier is significant because it* can help reduce brain cholinergic activity by inhibiting AChE and/or BChE activity [56]. Despite the in silico pharmacokinetic potential these 6 phytocompounds exhibit, their efficacies in both in vitro and in vivo pharmacokinetic settings need to be investigated.

## Conclusions

Collectively, the promising results from the drug-likeness analysis, binding affinity, and ADMET profile of the 6 phytocompounds (oxopowelline, andrographolide, deacetylbowdensine, 11, 12-dimethyl sageone, sageone, and quercetin) isolated from four Nigerian plants reported in the present study reveal that either the phytocompounds or the four plants (*Crinum jagus*, *Andrographis paniculata*, Sage plants (*Salvia officinalis L.*), and *Anacardium occidentale*) could be explored as potential antiviral agents to inhibit SARS-CoV-2 nsp16. However, in vitro and in vivo studies would have to be carried out to validate our findings.

## Supplementary Information

**Additional file 1: Supplementary Table 1.** Drug-likeness screening of phytocompounds from Nigerian based plants with antiviral properties.

## Data Availability

We declare that all the data generated are included in this study.

## Abbreviations

SARS-CoV 2: Severe acute respiratory syndrome coronavirus type 2
HCoV: Human coronavirus
Nsp16: Non-structural proteins
ADMET: Absorption, distribution, metabolism, excretion, and toxicity
BE: Binding energy
COVID-19: Coronavirus disease 19
AChE: Acetylcholinesterase
BChE: Butyrylcholinesterase
SFG: Sinefungin
