# Supplementary material for: Molecular docking and pharmacokinetic studies of phytocompounds from Nigerian Medicinal Plants as promising inhibitory agents against SARS-CoV-2 methyltransferase (nsp16)
Source: J Genet Eng Biotechnol. 2021 Nov 9;19:172. doi: 10.1186/s43141-021-00273-5 (PMC8576800; doi:10.1186/s43141-021-00273-5)
Supplement: Supplementary file 1 — Additional file 1: Supplementary Table 1. Drug-likeness screening of phytocompounds from Nigerian based plants with antiviral properties. [file 43141_2021_273_MOESM1_ESM.docx]

Supplementary information

Supplementary table 1. Drug-likeness screening of phytocompounds from Nigerian based plants with antiviral properties

| Phytocompound | MW | Lipinski violations | Ghose violations | Veber violations | Egan violations | Muegge violations | Bioavailability Score |
| --- | --- | --- | --- | --- | --- | --- | --- |
| Safficinollide | 344.4 | 0 | 0 | 0 | 0 | 0 | 0.55 |
| Sageone | 300.39 | 0 | 0 | 0 | 0 | 0 | 0.55 |
| 11,12-dimethyl sageone | 328.45 | 0 | 0 | 0 | 0 | 1 | 0.55 |
| Verticillatine A | 201.26 | 0 | 0 | 0 | 0 | 0 | 0.55 |
| Verticillatine B | 404.37 | 2 | 1 | 1 | 1 | 4 | 0.11 |
| Scandoside methyl ester | 414.36 | 1 | 1 | 1 | 1 | 3 | 0.11 |
| B-phenethylamine | 121.18 | 0 | 2 | 0 | 0 | 2 | 0.55 |
| Hypaphorine | 246.3 | 0 | 0 | 0 | 0 | 0 | 0.55 |
| Vasicine | 188.23 | 0 | 0 | 0 | 0 | 1 | 0.55 |
| Vasicinone | 202.21 | 0 | 0 | 0 | 0 | 0 | 0.55 |
| Vasicinol | 204.23 | 0 | 0 | 0 | 0 | 0 | 0.55 |
| Cryptolepine, | 232.28 | 0 | 0 | 0 | 0 | 0 | 0.55 |
| Malvalic acid | 280.45 | 1 | 1 | 1 | 0 | 1 | 0.85 |
| Sterculic Acid | 294.47 | 1 | 1 | 1 | 1 | 1 | 0.85 |
| 5,7-dihydroxy-3-isoprenyl flavones | 366.53 | 0 | 0 | 0 | 0 | 0 | 0.56 |
| 5-dihydroxy-3-isoprenyl flavones | 306.36 | 0 | 0 | 0 | 0 | 1 | 0.55 |
| 20-hydroxyecdysone | 494.66 | 1 | 3 | 0 | 1 | 1 | 0.55 |
| 20-hydroxy-(25-acetyl)-ecdysone-3-O-β-D-glucopyranoside, | 696.87 | 3 | 3 | 1 | 1 | 4 | 0.17 |
| Sidasterone A | 492.64 | 1 | 3 | 0 | 1 | 1 | 0.55 |
| Sidasterone B | 492.64 | 1 | 3 | 0 | 1 | 1 | 0.55 |
| S-(+)-N b-methyltryptophan methyl ester | 232.28 | 0 | 0 | 0 | 0 | 0 | 0.55 |
| 5`-hydroxymethyl-1`-(1,2,3,9-tetrahydro-pyrrolo [2, 1-b] quinazoline-1-yl)-hepta-1-one) | 300.4 | 0 | 0 | 0 | 0 | 0 | 0.55 |
| Licochalcone A | 338.4 | 0 | 0 | 0 | 0 | 0 | 0.55 |
| Licochalcone E | 324.37 | 0 | 0 | 0 | 0 | 0 | 0.55 |
| Glabridin | 215.29 | 0 | 0 | 0 | 0 | 0 | 0.55 |
| Glycyrrhetinic acid | 470.68 | 1 | 3 | 0 | 1 | 1 | 0.85 |
| Liquiritigerin | 256.25 | 0 | 0 | 0 | 0 | 0 | 0.55 |
| Androlycorine | 251.28 | 0 | 0 | 0 | 0 | 0 | 0.55 |
| Dihydrolycorine | 289.33 | 0 | 0 | 0 | 0 | 0 | 0.55 |
| Vittatine | 271.31 | 0 | 0 | 0 | 0 | 0 | 0.55 |
| 8-O-demethylmaritidine | 273.33 | 0 | 0 | 0 | 0 | 0 | 0.55 |
| Powelline | 301.34 | 0 | 0 | 0 | 0 | 0 | 0.55 |
| **Oxopowelline** | 299.32 | 0 | 0 | 0 | 0 | 0 | 0.55 |
| Buphanidrine | 315.36 | 0 | 0 | 0 | 0 | 0 | 0.55 |
| Galanthamine | 287.35 | 0 | 0 | 0 | 0 | 0 | 0.55 |
| Sanguinine | 273.33 | 0 | 0 | 0 | 0 | 0 | 0.55 |
| Narwedine | 285.34 | 0 | 0 | 0 | 0 | 0 | 0.55 |
| **Deacetylbowdensine** | 319.35 | 0 | 0 | 0 | 0 | 0 | 0.55 |
| Undulatine | 331.36 | 0 | 0 | 0 | 0 | 0 | 0.55 |
| Galanthamine-N-Oxide | 303.35 | 0 | 0 | 0 | 0 | 0 | 0.55 |
| Lycorine | 287.31 | 0 | 0 | 0 | 0 | 0 | 0.55 |
| **Andrographolide** | 318.45 | 0 | 0 | 0 | 0 | 0 | 0.55 |
| Andrograpanin | 306.27 | 1 | 0 | 0 | 0 | 1 | 0.55 |
| Gallocatechin | 344.27 | 1 | 1 | 1 | 1 | 2 | 0.11 |
| 4-O-Galloylquinic acid | 634.45 | 3 | 2 | 1 | 1 | 4 | 0.17 |
| Corilagin | 634.45 | 3 | 2 | 1 | 1 | 4 | 0.17 |
| Isocorilagin | 650.45 | 3 | 3 | 1 | 1 | 5 | 0.17 |
| Phyllanthine | 217.26 | 0 | 0 | 0 | 0 | 0 | 0.55 |
| Securinine | 221.25 | 0 | 0 | 0 | 0 | 0 | 0.55 |
| Isobubbialine | 221.25 | 0 | 0 | 0 | 0 | 0 | 0.55 |
| Epibubbialine | 203.24 | 0 | 0 | 0 | 0 | 0 | 0.55 |
| Nor securinine | 456.7 | 1 | 3 | 0 | 1 | 1 | 0.85 |
| Oleanolic acid | 456.7 | 1 | 3 | 0 | 1 | 1 | 0.85 |
| Ursolic acid | 154.25 | 0 | 1 | 0 | 0 | 2 | 0.55 |
| Linalool | 474.37 | 2 | 0 | 2 | 1 | 3 | 0.11 |
| Amarosterol A | 298.46 | 1 | 0 | 0 | 0 | 2 | 0.55 |
| Amarosterol B | 428.73 | 1 | 3 | 0 | 1 | 2 | 0.55 |
| Phyllanthenol | 456.74 | 1 | 3 | 0 | 1 | 1 | 0.55 |
| Phyllantheol | 428.73 | 1 | 3 | 0 | 1 | 2 | 0.55 |
| Lupeol | 426.72 | 1 | 3 | 0 | 1 | 2 | 0.55 |
| Ellagic Acid | 302.19 | 0 | 0 | 1 | 1 | 0 | 0.55 |
| Gallic acid | 170.12 | 0 | 2 | 0 | 0 | 1 | 0.56 |
| Phytol | 296.53 | 1 | 1 | 1 | 1 | 2 | 0.55 |
| Cichoric Acid | 412.69 | 1 | 3 | 0 | 1 | 2 | 0.55 |
| Chlorogenic Acid | 354.31 | 1 | 1 | 1 | 1 | 2 | 0.11 |
| Caffeic Acid | 180.16 | 0 | 0 | 0 | 0 | 1 | 0.56 |
| Nitidarin diisovalerianate | 568.7 | 1 | 4 | 1 | 1 | 1 | 0.55 |
| Stigmasterol | 204.35 | 1 | 0 | 0 | 0 | 1 | 0.55 |
| (E)-caryophyllene | 576.85 | 1 | 4 | 0 | 0 | 1 | 0.55 |
| Sitosterol 3-O-β-galactopyranoside | 414.71 | 1 | 3 | 0 | 1 | 2 | 0.55 |
| Sitosterol | 204.35 | 1 | 0 | 0 | 0 | 1 | 0.55 |
| Germacrene D | 448.38 | 2 | 0 | 1 | 1 | 3 | 0.17 |
| Quercetin 3-O-rhamnoside | 470.68 | 1 | 3 | 0 | 1 | 1 | 0.85 |
| **Quercetin** | 302.24 | 0 | 0 | 0 | 0 | 0 | 0.55 |
| Isoquercetrin | 464.38 | 2 | 1 | 1 | 1 | 3 | 0.17 |
| Rutin | 610.52 | 3 | 4 | 1 | 1 | 4 | 0.17 |
| Deacetylkhayanolide E | 516.54 | 1 | 1 | 1 | 1 | 1 | 0.55 |
| Khayanolide A | 516.54 | 1 | 1 | 1 | 1 | 1 | 0.55 |
| 6-Phenyl,4-(1’oxyethylphenyl) hexane | 266.38 | 1 | 0 | 0 | 0 | 2 | 0.55 |
| Benzene 1,1’-(oxydiethylidene) bis | 226.31 | 0 | 0 | 0 | 0 | 1 | 0.55 |
| Carbamic acid, 4-methyl-1-phenyl)-1-phenyl | 255.31 | 0 | 0 | 0 | 0 | 0 | 0.55 |
| 3,4-Epoxyclerodan-13E-en-15-oic acid | 320.47 | 0 | 0 | 0 | 0 | 1 | 0.85 |
| 5α,8α-(2-oxokolavenic acid | 318.45 | 0 | 0 | 0 | 0 | 0 | 0.85 |
| Copalic acid | 304.47 | 1 | 0 | 0 | 0 | 1 | 0.85 |
| 3,4-dihydroclerodan-13z-en-15-oic acid | 318.49 | 1 | 1 | 0 | 0 | 1 | 0.85 |
| 3,4-dihydroxyclerodan-13E-en-15-oic acid | 366.53 | 0 | 0 | 0 | 0 | 0 | 0.56 |
| Oxokolavemic acid | 346.5 | 0 | 0 | 0 | 0 | 1 | 0.85 |
| 3,4-Dimethoxyphenol β-D-apiofuranosyl(1′->6′)-β-D-glucopyranoside | 478.44 | 2 | 1 | 1 | 1 | 4 | 0.17 |
| Procyanidin B2 | 578.52 | 3 | 2 | 1 | 1 | 3 | 0.17 |
| 2β-methoxyclovan-9α-ol | 252.39 | 0 | 0 | 0 | 0 | 0 | 0.55 |
| Methyl-ent-3β-hydroxylabd-8(17)-en-15-oate | 338.52 | 0 | 0 | 0 | 0 | 1 | 0.55 |
| Clovane-2β,9α-diol | 238.37 | 0 | 0 | 0 | 0 | 0 | 0.55 |
| Alepterolic acid | 322.48 | 0 | 0 | 0 | 0 | 0 | 0.55 |
| Anticopalic acid | 306.48 | 1 | 1 | 0 | 1 | 1 | 0.55 |
| (3S,5R,6S)-trihydroxy-7E-megastigmen-9-one | 244.33 | 0 | 0 | 0 | 0 | 0 | 0.55 |
| β-amyrin | 428.73 | 1 | 3 | 0 | 1 | 2 | 0.55 |
| Piliostigmin | 328.32 | 0 | 0 | 0 | 0 | 0 | 0.55 |
| Vitamin E | 430.71 | 1 | 3 | 1 | 1 | 1 | 0.55 |
| 3-hexenyl-1-O β-D-glucopyranoside | 262.3 | 0 | 1 | 0 | 0 | 0 | 0.55 |
| Anthocyanidin alkaloid | 241.2 | 0 | 0 | 0 | 0 | 0 | 0.55 |
